# Insulin clearance and incretin hormones following oral and “isoglycemic” intravenous glucose in type 2 diabetes patients under different antidiabetic treatments

**DOI:** 10.1038/s41598-022-06402-5

**Published:** 2022-02-15

**Authors:** Andrea Tura, Christian Göbl, Irfan Vardarli, Giovanni Pacini, Michael Nauck

**Affiliations:** 1 CNR Institute of Neuroscience, Corso Stati Uniti 4, 35127 Padova, Italy; 2 Department of Obstetrics and Gynaecology, Medical University of Vienna, Vienna, Austria; 3 Diabetes Division, Katholisches Klinikum Bochum, St. Josef Hospital (Ruhr University Bochum), Bochum, Germany; 4 Independent Researcher, Padova, Italy

**Keywords:** Type 2 diabetes, Metabolism

## Abstract

It has not been elucidated whether incretins affect insulin clearance in type 2 diabetes (T2D). We aimed exploring possible associations between insulin clearance and endogenously secreted or exogenously administered incretins in T2D patients. Twenty T2D patients were studied (16 males/4 females, 59 ± 2 years (mean ± standard error), BMI = 31 ± 1 kg/m^2^, HbA1c = 7.0 ± 0.1%). Patients were treated with metformin, sitagliptin, metformin/sitagliptin combination, and placebo (randomized order). On each treatment period, oral and isoglycemic intravenous glucose infusion tests were performed (OGTT, IIGI, respectively). We also studied twelve T2D patients (9 males/3 females, 61 ± 3 years, BMI = 30 ± 1 kg/m^2^, HbA1c = 7.3 ± 0.4%) that underwent infusion of GLP-1(7–36)-amide, GIP, GLP-1/GIP combination, and placebo. Plasma glucose, insulin, C-peptide, and incretins were measured. Insulin clearance was assessed as insulin secretion to insulin concentration ratio. In the first study, we found OGTT/IIGI insulin clearance ratio weakly inversely related to OGTT/IIGI total GIP and intact GLP-1 (R^2^ = 0.13, *p* < 0.02). However, insulin clearance showed some differences between sitagliptin and metformin treatment (*p* < 0.02). In the second study we found no difference in insulin clearance following GLP-1 and/or GIP infusion (*p* > 0.5). Thus, our data suggest that in T2D there are no relevant incretin effects on insulin clearance. Conversely, different antidiabetic treatments may determine insulin clearance variations.

## Introduction

Plasma insulin concentration is determined by a balance between the amount of insulin secreted by pancreatic beta cells and that eliminated from the circulation, mainly by the liver, but also by the kidneys and other tissues^1^. Thus, changes in plasma insulin can be due to alterations in insulin secretion, but also in insulin clearance. In short, insulin clearance can be described as the disappearance of insulin from the bloodstream of the entire organism, and it can be conceptualized as the sum of two main processes, *i.e.*, hepatic clearance and extrahepatic clearance^2^. The interest for the study of insulin clearance has recently increased as it has become evident that insulin clearance is a highly regulated process relevant for the maintenance of glucose homeostasis^2^. Recent studies also suggested that insulin clearance is altered in subjects with type 2 diabetes^3^, as well as other types of diabetes^4,5^. Of note, alterations in insulin clearance may appear already in the prediabetes states, showing a tendency to decrease when subjects progress towards dysglycemia and possibly type 2 diabetes^6^. In addition, it has been shown that insulin clearance is not only involved in glucose homeostasis, but also linked to other pathophysiological conditions, such as atherosclerosis or other cardiovascular abnormalities^7–10^.

There is also evidence that insulin clearance is reduced following oral compared to intravenous glucose administration. This was shown in some pioneering studies already some decades ago, with an emphasis on changes in hepatic insulin clearance^11–15^. However, only one of those studies included patients with type 2 diabetes^15^. In fact, even in more recent studies, the quantification of insulin clearance reduction after oral *vs.* intravenous glucose administration in type 2 diabetes has not been extensively examined^16,17^.

On the other hand, some studies have suggested a possible effect of incretin hormones on insulin clearance^18–21^, and this may explain the clearance reduction in oral *vs.* intravenous glucose challenge, since incretin hormone concentrations typically rise after oral, but not after intravenous glucose administration^22^. However, the degree of the possible association of such insulin clearance reduction to the incretin hormones increase has not been clearly investigated. In addition, previous studies^18–21^ did not include participants with type 2 diabetes.

The main aim of this investigation was therefore to quantify the reduction in insulin clearance following an oral glucose tolerance test compared to an isoglycemic intravenous glucose infusion in participants with type 2 diabetes (treated with metformin, sitagliptin, metformin/sitagliptin combination, or placebo) and to explore the possible association between the reduction in insulin clearance and the rise in incretin hormones levels. We also analyzed the possible effect of the indicated antidiabetic treatments on insulin clearance. Furthermore, we corroborated our findings with a second analysis in participants with type 2 diabetes that underwent intravenous high-dose infusions of GLP-1(7–36)-amide, GIP, GLP-1/GIP combination, or placebo.

## Methods

### Participants

In the first study (Study 1), we included twenty patients with type 2 diabetes. The main characteristics of the patients were described in detail in a previous study^23^. Briefly, inclusion criteria were (a) no antidiabetic medication, except metformin or sulfonylurea (monotherapy); (b) age in 30–75 years range; (c) BMI in 25–35 kg/m^2^ range; (d) glycated hemoglobin (HbA1c) ≥ 6.5% (48 mmol/mol) and ≤ 9.0% (75 mmol/mol) if drug-naïve, or ≥ 6.0% (42 mmol/mol) and ≤ 8.5% (69 mmol/mol) if treated with metformin or sulfonylurea; (e) fasting plasma glucose ≥ 6.1 mmol/L and ≤ 12.2 mmol/L before and after 2-week placebo run-in period; (f) male or non-fertile female. The eligible patients were 16 males and 4 females, were 59 ± 2 years old (mean ± standard error, SE), and had a BMI of 31 ± 1 kg/m^2^. HbA1c was 7.0 ± 0.1% (53 ± 1 mmol/mol), with a known diabetes duration of 5 ± 1 years. In the second study (Study 2), we included patients with basal insulin-treated type 2 diabetes, whose detailed characteristics were reported in a previous study^24^. The patients were 9 males and 3 females, were 61 ± 3 years old, and had a BMI of 30 ± 1 kg/m^2^; HbA1c was 7.3 ± 0.4%, (56 ± 4 mmol/mol) and known diabetes duration was 7 ± 2 years.

### Study design, procedures and measures

Both study protocols were approved by the Georg August University Göttingen Ethics Committee (October 2008, registration number 1/4/08, and June 2008, registration number 14/3/01, respectively). Written informed consent was obtained from all participants in both studies. All research procedures were performed in accordance to the relevant guidelines and regulations.

In the Study 1 (see Supplementary Fig. S1 online), patients entered a 6-week washout period if previously treated with oral antidiabetic agents. After 2-week single blind run-in period, patients entered the crossover study period, based on four double blind treatment periods for 6 days each (order randomized) with 4-week washout period between treatments. Treatments consisted in sitagliptin (100 mg daily), metformin (from 500 to 2000 mg daily), metformin/sitagliptin combination, or placebo. The tests were performed after an overnight fast. On day 5 of each treatment period, a 75 g oral glucose tolerance test (OGTT) was performed, followed on day 6 by an isoglycemic intravenous glucose infusion (IIGI). In the IIGI test, the OGTT glucose profile was reproduced by a variable intravenous glucose administration. In both tests, blood samples were taken at basal and at 15, 30, 45, 60, 90, 120, 180, and 240 min. Plasma glucose, insulin, C-peptide, total GLP-1 and total GIP (C-terminally directed assay), intact GLP-1 (sandwich ELISA) and intact GIP (N-terminally directed assay) were determined as previously described^25^.

In the Study 2 (see again Supplementary Fig. S1), patients underwent a screening examination and four tests on different days in randomized order. Antidiabetic medications were discontinued one day before each experiment. Experiments were started in the morning after an overnight fast. Either placebo (vehicle: 0.9% NaCl with 1% human serum albumin), GLP-1(7–36)-amide (1.2 pmol kg^-1^ min^-1^), GIP (4 pmol kg^-1^ min^-1^), or a combination of both incretin hormones was infused over 360 min. This dose of GLP-1 was previously proven to normalize fasting glycemia in patients with type 2 diabetes without side effects^26,27^. The dose of GIP was expected to result in clearly high, pharmacological concentrations. Safety of such dose was ensured by the fact that previous studies used even higher GIP doses^28^. The human synthetic GLP-1 and GIP (GMP grade, for human use) were obtained from PolyPeptide, Wolfenbüttel, Germany, and prepared for the administration to human subjects by Pegasus Pharma, Hannover, Germany. Plasma glucose, insulin, C-peptide, total and intact GLP-1 and GIP were determined as previously described^24^.

### Calculations

**Figure 1:**
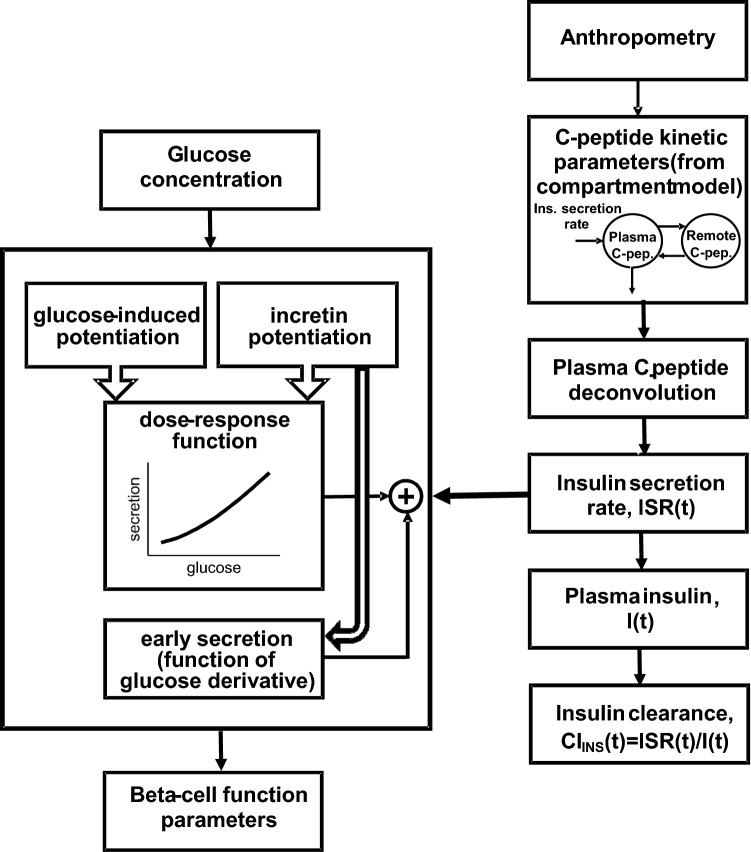
Block diagram of the mathematical modeling approach. The steps represented in the blocks on the right are repeated simultaneously for both OGTT and IIGI data, thus providing insulin secretion estimation from both tests for the assessment of the beta-cell function parameters (blocks on the left). ISR(t) is insulin secretion rate, I(t) is plasma insulin. CL_INS_(t) is obtained as the ratio of the former to the latter.

OGTT and IIGI tests were analyzed simultaneously using a mathematical model for the assessment of beta-cell function and, specifically, for the incretin hormone actions on insulin secretion^29,30^. A schematic diagram of the adopted procedure is displayed in Fig. 1. In brief, insulin secretion is associated with glucose concentration during the IIGI test through the basic mechanisms of a previous, classical model for the analysis of a single test, *i.e.*, a dose–response function, an early secretion component, and a glucose-induced potentiation term^31^. In the OGTT, the effect of incretins on insulin secretion includes two factors: a potentiation factor that acts on insulin secretion during the whole test duration, and an increase in early insulin secretion. The main model parameters are the beta-cell glucose sensitivity, G_SENS_ (mean slope of the beta-cell dose–response from the IIGI), the glucose-induced potentiation, P_GLU_(t), representing the modulation of the dose–response during IIGI, the incretin-induced potentiation, P_INCR_(t), quantifying the time course of the incretin effect, and the rate sensitivity, R_SENS_, determined separately in the OGTT and IIGI, quantifying early insulin secretion. From P_GLU_(t), the potentiation factor ratio, PFR, is derived as P_GLU_ at end of the test divided by P_GLU_ at basal. From P_INCR_(t), the average value during the test is computed, P_INCRm_. When higher than 1, P_INCRm_ indicates significant enhancement of insulin secretion attributed to the incretin effect. In addition, the time course of insulin secretion rate during both the OGTT and the IIGI are provided. Similarly, modeling analysis was also applied for the assessment of the insulin secretion rate in the experiments with intravenous administration of GLP-1, GIP, GLP-1/GIP combination, and placebo^24^. In both studies, insulin clearance rate was calculated as the ratio of insulin secretion rate to plasma insulin concentration pattern. Average insulin clearance during the OGTT and IIGI test was then computed. Insulin sensitivity was assessed in the Study 1 by PREDIM^32^. The area-under-the-curve (AUC) of the variables was calculated by the trapezoidal rule.

Modeling analysis was performed in MATLAB®. Parameter estimations were obtained by nonlinear least-squares fitting approach, through the *lsqnonlin* function. Both function and step-size tolerances have been set to 10^–6^, as done previously^33^.

### Statistical analyses

Difference in insulin clearance (as well as in other metabolic parameters) from the OGTT and the IIGI was tested by repeated measures ANOVA approach, since for both OGTT and IIGI four parameter values were available in each patient (relating to the four different treatments). Pairwise comparisons were used to assess possible variations in insulin clearance among treatments, both in the OGTT and in the IIGI, as well as possible variations in the insulin clearance difference between the two tests, expressed as the OGTT/IIGI insulin clearance ratio. One sample *t*-test allowed comparison of variables to a constant value (*i.e.*, 1).

Regression analyses were performed through linear mixed-effect model approach (whereby the patient’s identification number was included as a random effect) to assess possible associations between some metabolic parameters, especially the OGTT/IIGI insulin clearance ratio and the corresponding plasma incretin hormones ratios, with possible adjustments as appropriate in a multivariable design. Similar approach was used to assess possible associations in both OGTT and IIGI between the insulin clearance fold increase over basal (*i.e.*, OGTT or IIGI value normalized to the basal value) and similarly computed variables, in particular the plasma incretin hormones concentration. In the study with GLP-1, GIP, GLP-1/GIP combination and placebo experiments, possible differences between insulin clearance at basal and following the intravenous administration (in the 60–360 min interval) was assessed for each experiment by paired *t*-test.

Variable distributions were assessed before statistical testing, and logarithmic transformation was performed in case of skewed distributions. The two-sided significance was set at the 5% level (*p* < 0.05). Values are reported as mean ± SE.

## Results

### Insulin secretion, beta-cell function and insulin sensitivity

**Figure 2:**
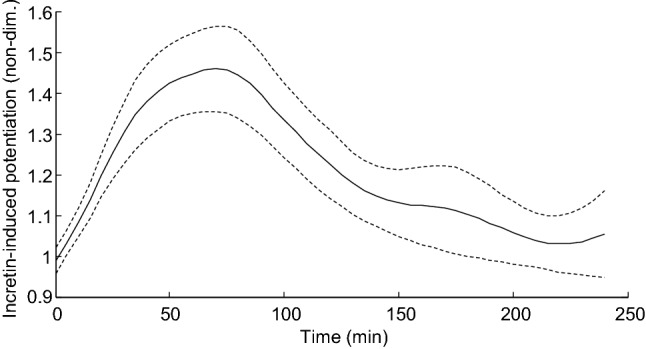
Time course of the incretin potentiation during the OGTT assessed by mathematical modeling (mean value: solid line; 95% confidence intervals: dashed lines).

In Table 1, plasma concentrations are reported for glucose, insulin, C-peptide and incretin hormones, for both OGTT and IIGI. Table 1 also reports the value of the insulin secretion/beta-cell function parameters for the two tests. Total insulin secretion (TIS), calculated as the AUC of insulin secretion rate during the test, was higher in OGTT than in IIGI (*p* < 0.0001), in agreement with the previous study by Nauck et al*.*^23^. This was confirmed by the incretin potentiation parameter, P_INCRm_, higher than 1 (*p* < 0.0001). The time course of P_INCR_(t) is reported in Fig. 2. In addition, R_SENS_ was higher in the OGTT (*p* < 0.0001). Insulin sensitivity was virtually the same in the two tests (*p* = 0.94, Table 1).

**Table 1:** Plasma concentration of glucose, insulin, C-peptide and incretin hormones, and model-derived parameters of insulin secretion, beta-cell function, and insulin sensitivity, for the OGTT and the isoglycemic intravenous glucose infusion test (IIGI), in the analyzed twenty patients with type 2 diabetes (Study 1).

	OGTT	IIGI
**Plasma glucose, insulin, C-peptide**		
Basal glucose (mmol/L)	7.31 ± 0.16	7.26 ± 0.15
Mean glucose (mmol/L)	11.23 ± 0.29	11.47 ± 0.30
Basal insulin (pmol/L)	94.4 ± 5.1	97.5 ± 5.1
Mean insulin (pmol/L)	343.6 ± 23.0	235.6 ± 16.2 ^‡^
Basal C-peptide (pmol/L)	699 ± 21	717 ± 21
Mean C-peptide (pmol/L)	1594 ± 55	1314 ± 47 ^‡^
**Plasma GLP-1 and GIP**		
Basal total GLP-1 (pmol/L)	9.21 ± 0.55	9.49 ± 0.61
Mean total GLP-1 (pmol/L)	11.88 ± 0.63	8.45 ± 0.44 ^‡^
Basal total GIP (pmol/L)	16.53 ± 1.24	15.59 ± 0.90
Mean total GIP (pmol/L)	39.34 ± 1.79	12.29 ± 0.67 ^‡^
Basal intact GLP-1 (pmol/L)	4.13 ± 0.39	3.97 ± 0.36
Mean intact GLP-1 (pmol/L)	4.96 ± 0.37	3.17 ± 0.35 ^‡^
Basal intact GIP (pmol/L)	23.79 ± 0.95	20.90 ± 0.81
Mean intact GIP (pmol/L)	35.99 ± 1.29	20.40 ± 0.67 ^‡^
**Insulin secretion**		
Basal insulin secretion, ISR_b_ (pmol min^-1^ m^-2^)	88.21 ± 2.62	89.74 ± 2.55
Total insulin secretion, TIS (nmol m^-2^)	51.49 ± 1.92	42.75 ± 1.64 ^‡^
**Beta-cell function**		
Glucose sensitivity, G_SENS_ (pmol min^-1^ m^-2^ mM^-1^) ^†^	15.72 ± 1.66	
Rate sensitivity, R_SENS_ (pmol m^-2^ mM^-1^)	223.7 ± 29.8	44.7 ± 10.2 ^‡^
Glucose-induced potentiation factor ratio, PFR (non-dimensional) ^†^	1.46 ± 0.05	
Average incretin-induced potentiation, P_INCRm_ (non-dimensional)	1.21 ± 0.03	-
**Insulin sensitivity**		
PREDIM (mg kg^-1^ min^-1^)	2.51 ± 0.07	2.56 ± 0.07

Data are reported as mean ± SE.

^†^The same value in OGTT and IIGI (by model hypothesis); ^‡^ significant difference between OGTT and IIGI (by repeated measures ANOVA).

### Insulin clearance variations and relationships

**Figure 3:**
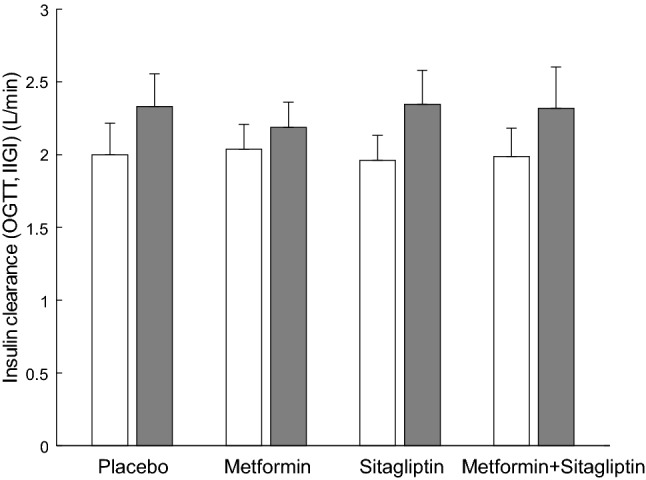
Insulin clearance (mean ± SE bars) in OGTT (white) and IIGI (grey), for the four different treatments (placebo, metformin, sitagliptin, combination of metformin and sitagliptin), in Study 1.

In the Study 1, insulin clearance during OGTT was significantly lower than during IIGI (2.0 ± 0.1 *vs.* 2.3 ± 0.1 L/min, respectively, *p* < 0.0001). Figure 3 reports insulin clearance for OGTT and IIGI separately for the four treatments. The difference in insulin clearance between the two tests was confirmed in all four treatments (*p* < 0.002).

Such difference in insulin clearance between the two tests, expressed as ratio of OGTT insulin clearance to IIGI insulin clearance (*i.e.*, CL_INS_OGTT_/CL_INS_IIGI_), was weakly inversely related to OGTT/IIGI total insulin secretion ratio (*i.e.*, TIS_OGTT_/TIS_IIGI_), after adjusting for the different treatments in the regression analysis (R^2^(marginal) = 0.15, *p* < 0.009). Consistently, similar results were found for regression between OGTT/IIGI insulin clearance ratio and P_INCRm_ (R^2^ = 0.16, *p* < 0.005).

When analyzing possible associations with plasma incretin hormones levels, we found that the CL_INS_OGTT_/CL_INS_IIGI_ ratio was weakly inversely related to the OGTT/IIGI ratio of the total GIP AUC (R^2^ = 0.13, *p* < 0.02). Intact GIP AUC ratio, and both total and intact GLP-1 ratios, were unrelated to CL_INS_OGTT_/CL_INS_IIGI_ ratio (*p* > 0.11). When including both GLP-1 and GIP in the analysis (separately for total and intact forms), a relationship emerged between CL_INS_OGTT_/CL_INS_IIGI_ ratio and intact GLP-1 ratio, but still weak (again, R^2^ = 0.13, *p* < 0.02). As regards possible relationships at single time instants, CL_INS_OGTT_/CL_INS_IIGI_ ratio was inversely related to OGTT/IIGI total GIP and intact GLP-1 ratios at few instants only (fasting and 15 min for the former (*p* < 0.01), 30 and 60 min for the latter (*p* < 0.03)).

**Figure 4:**
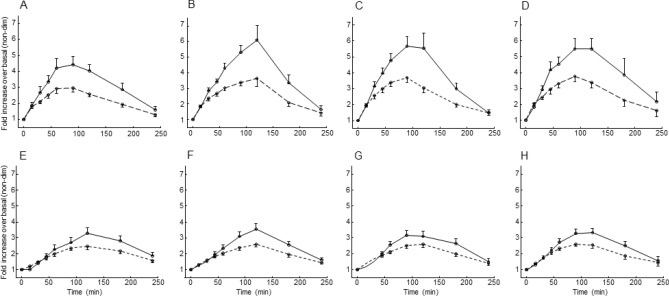
Fold increments over basal in plasma insulin concentrations (solid line) and insulin secretion rates (dashed line) in OGTT (upper panels) and IIGI (lower panels), for the four different treatments (placebo (**A**, **E**), metformin (**B**, **F**), sitagliptin (**C**, **G**), combination of metformin and sitagliptin (**D**, **H**)), in Study 1 (mean ± SE bars).

**Figure 5:**
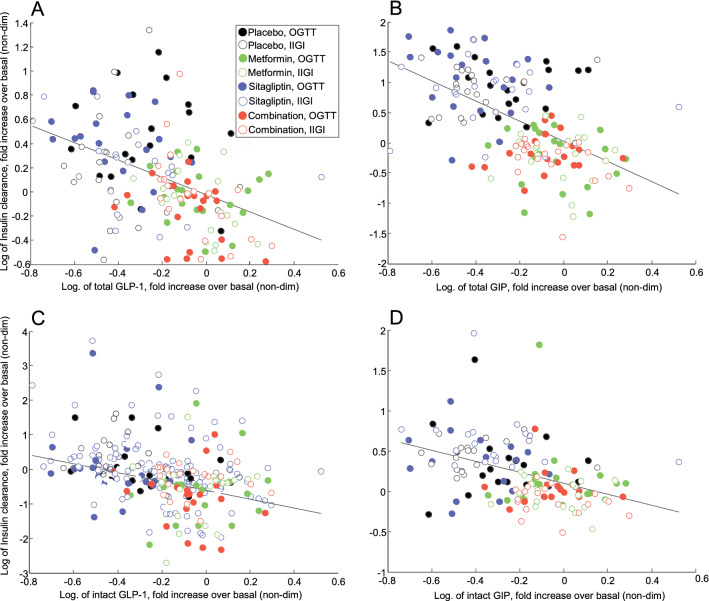
Regression plots relating fold increments over basal of total GLP-1 and GIP levels (**A**, **B**), as well as intact GLP-1 and GIP levels (**C**, **D**), to fold increments over basal of insulin clearance, in OGTT and IIGI and for the four different treatments (placebo, metformin, sitagliptin, combination of metformin and sitagliptin), in Study 1. Values are logarithmically transformed.

We also analyzed possible associations between insulin clearance fold increase over basal and similarly computed variables. OGTT and IIGI insulin secretion rate and plasma insulin concentration fold increase for the different treatments are reported in Fig. 4. When considering values from both OGTT and IIGI in all treatments, the derived insulin clearance fold increase showed association with both plasma GLP-1 and GIP, both total and intact forms (*p* < 0.0001), as shown in Fig. 5. However, even in this analysis the relationships were not strong, (average R^2^ = 0.20), and, especially, relationships were typically lost following adjustment for insulin secretion (remaining significant only for total GLP-1), suggesting that lower insulin clearance for higher incretin hormones levels may be due to higher insulin secretion, rather than to a direct effect of incretins.

**Figure 6:**
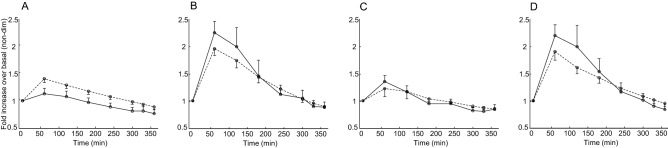
Fold increments over basal in plasma insulin concentrations (solid line) and insulin secretion rates (dashed line) for the four different exogenous intravenous administrations (placebo (**A**), GLP-1 (**B**), GIP (**C**), combination of GLP-1 and GIP (**D**)), in Study 2 (mean ± SE bars).

**Figure 7:**
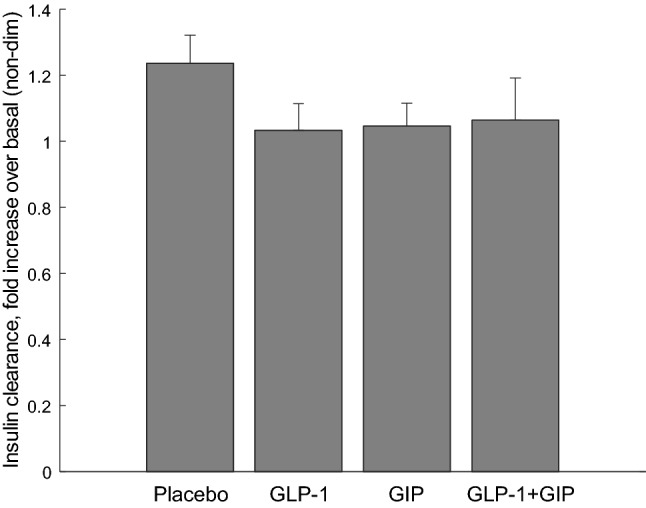
Fold increments over basal of insulin clearance during 60–360 min following exogenous intravenous administration of placebo, GLP-1, GIP, or combination of GLP-1 and GIP, in Study 2 (mean ± SE bars).

In the Study 2, we again calculated insulin secretion rate and plasma insulin concentration fold increase over basal. Figure 6 reports their temporal patterns for the different experiments, based on exogenous intravenous administration of placebo, GLP-1, GIP, or combination of GLP-1 and GIP. When comparing basal insulin clearance to average post-stimulus insulin clearance (60–360 min interval), we did not find significant increase following either GLP-1 or GIP, or their combination (*p* ≥ 0.52; see Fig. 7).

### Differences in insulin clearance among antidiabetic treatments

From the data of the Study 1, we also analyzed possible differences in insulin clearance between metformin, sitagliptin, metformin and sitagliptin combination, and placebo treatment. For both OGTT and IIGI insulin clearance, we did not find differences among treatments (*p* > 0.15). However, CL_INS_OGTT_/CL_INS_IIGI_ ratio was found slightly lower after sitagliptin compared to metformin (0.86 ± 0.03 *vs.* 0.93 ± 0.02, *p* < 0.02).

## Discussion

In this study, we found that insulin clearance reduction in OGTT *vs.* IIGI shows some associations to plasma incretin levels. However, the associations were significant only for total GIP and for intact GLP-1 (specifically, their OGTT/IIGI ratios) in multivariable statistical analysis, and, more importantly, the associations were weak. This suggests that the effect of incretins on insulin clearance is small or negligible, and other factors should have a higher impact in determining the insulin clearance reduction following OGTT *vs.* IIGI.

Some studies showed that insulin clearance (at least its hepatic component) is reduced following oral rather than intravenous glucose administration, thus suggesting a possible effect of incretin hormones in insulin clearance reduction between the two routes of glucose administration^11–17^. However, only few of them included patients with type 2 diabetes^15–17^, and this motivated us to carry out the present investigation. In addition, some studies supported the hypothesis of an effect of incretins on insulin clearance based on exogenous administration of GIP^18^ or GLP-1 receptor agonist^19^, but other studies showed different findings^20^, ^21^. Thus, the possible effect of incretins on insulin clearance is still a matter of debate. Furthermore, it is worth noting that some of the indicated studies^18–21^ were based on exogenous administration of incretin hormones (or receptor agonist), whereas the other studies^11–17^ did not specifically investigate the possible associations of insulin clearance and incretin levels. The main aim of our investigation was therefore to assess possible associations between insulin clearance reduction following oral *vs.* intravenous glucose administration and endogenous plasma incretin concentrations, *i.e.*, at the physiological levels. To our knowledge, no previous studies reported similar data. In addition, we also analyzed data related to incretin hormones exogenous administration, similarly to some of the previous studies^18–21^.

Since the effect of incretins on insulin clearance appears small, question arises as to what factors are the main determinants of the insulin clearance reduction following OGTT *vs.* IIGI. Such factors may be related to the higher insulin levels in OGTT *vs.* IIGI, in particular the saturation of the hepatic insulin extraction. Of note, some studies in subjects undergoing hepatic vein or renal vein catheterization reported hepatic insulin extraction reaching saturation only at high insulin concentration values, *i.e.*, about 500 µU/ml in the hepatic artery (which however can be assumed similar to mixed venous blood concentration)^34^. Such insulin concentration values were not reached in our data, but it should be noted that, at the indicated insulin concentration, the hepatic insulin extraction was reported to drop to half the value observed at lower (physiological) concentration (from about 60% to 30%)^34^. Thus, it is possible that the hepatic insulin extraction saturation starts at insulin concentration values still in the physiological range, and hence the phenomenon may have occurred in our experiments. This may explain the OGTT to IIGI insulin clearance reduction. Notably, this hypothesis is also supported by the results of our previous study in subjects undergoing measurement of insulin and C-peptide in the femoral artery and hepatic vein by means of the hepatic catheterization technique^35^. Though statistically significant difference in hepatic insulin extraction was found only in diabetic *vs.* control subjects and not *vs.* obese subjects, in general we observed a clear tendency to reduced hepatic insulin extraction for higher plasma insulin levels^35^.

It should be noted that plasma incretin hormones levels may not be good markers of their effect^29,30^. In fact, though it is widely recognized that incretin hormones typically enhance insulin secretion, in both subjects with and without type 2 diabetes poor association was described between the insulin secretion increase following OGTT compared to IIGI and related incretin hormones levels, both as average values in the studied populations and in terms of individual relationships^29^. On the other hand, the degree of association between insulin secretion enhancement and incretin hormones levels was typically not negligible (observed in about 20% of the tests^29^, and, especially, higher than what we observed here between insulin clearance and incretin levels, both in terms of AUCs and of individual relationship. In fact, in the present analysis no more than 3% of the tests showed individual relationship between OGTT insulin clearance and incretin hormone levels, with GIP performing even worse than GLP-1 (not shown).

To corroborate our findings on the possible effect of incretin hormones on OGTT to IIGI insulin clearance reduction, we studied the possible variations of insulin clearance following exogenous infusion of GLP-1, GIP, or a combination of both (*i.e.*, in this case administering incretin hormones at supraphysiological, pharmacological levels). Interestingly, we found that basal and post-stimulus insulin clearance values were extremely similar following the administration of either GLP-1 or GIP, or their combination. This enforces the conclusion that in type 2 diabetic patients the effect of incretin hormones on insulin clearance appears negligible.

Some studies analyzed the possible effect of incretin hormones on insulin clearance in animal models^36–38^. In those studies, mice with double^36,37^ or single^38^ incretin receptor knockout were compared to wild-type mice, with normal activity of the incretin hormones receptors. In mice with single receptor knockout no differences were observed in insulin clearance compared to wild-type mice^38^. Instead, in mice with double incretin receptor knockout an increase in insulin clearance was observed in the first phase of the experiment based on intravenous glucose administration, this suggesting a possible effect on insulin clearance of the incretin hormones even at basal conditions^36^. In addition, it was shown that the difference in insulin clearance between double incretin receptor knockout mice and wild-type mice appears related to the peripheral rather than the hepatic insulin clearance component^37^. In our opinion, differences in the physiology of mice compared to humans, as well as in the type of glucose stimulation performed in the studies on double incretin receptor knockout mice^36,37^ compared to the present study, may explain the conflicting findings between those studies in animals and this study. It is also worth noting that future studies may benefit from larger samples size and, possibly, from metabolic tests with different doses of glucose, as done in previous studies^30^.

A secondary aim of our investigation was assessing the possible effects on insulin clearance of the antidiabetic treatments that participants underwent. Indeed, few studies addressed such issue^39–43^, and to our knowledge none in subjects undergoing different antidiabetic treatments. In more details, insulin clearance tended to increase following treatment with liraglutide^39^. Insulin clearance was found increased also with sodium glucose co-transporter 2 inhibitors: specifically, canagliflozin increased the total insulin clearance^40^, whereas dapagliflozin increased its hepatic component^41^. Dapagliflozin increased insulin clearance also in the study by Ekholm et al.^42^, both as single treatment or in combination with saxagliptin. However, there was no difference in insulin clearance with saxagliptin alone, and similar results were found with sitagliptin in one of our previous studies^43^. Accordingly, in the present study we did not find any difference in insulin clearance with sitagliptin, when compared to placebo (neither from OGTT nor IIGI). Similarly, no differences were observed with metformin compared to placebo. However, interestingly we found a slight reduction in insulin clearance with sitagliptin compared to metformin, at least when considering the OGTT/IIGI insulin clearance ratio. These findings suggest that the possible effect of the different antidiabetic agents on insulin clearance may vary (some agents determining an increase in insulin clearance, whereas other agents determining a reduction). On the other hand, the number of studies on the effect of the antidiabetic agents on insulin clearance is still limited, and hence such issue should be further investigated in studies with appropriate specific primary endpoint. Indeed, this may be important for optimal individual dosing of the different antidiabetic agents, in agreement with the recommendations for precision therapeutics^44^.

In conclusion, this study presents for the first time a detailed analysis of the possible effect of the incretin hormones on insulin clearance in patients with type 2 diabetes. Based on our data, the weak associations between the OGTT to IIGI insulin clearance reduction and the plasma incretin hormones increase, and the lack of variation in insulin clearance following exogenous incretin administration, does not suggest existence of causal relationship between these variables. Thus, our study indicates in type 2 diabetes the absence of relevant effects of incretin hormones on insulin clearance. Finally, our study suggests that different antidiabetic treatments may determine heterogeneous effects on insulin clearance, and this deserves further insight, especially in the context of optimal pharmacological dosing.

## Supplementary Information

Supplementary Information.

## Data Availability

The datasets analyzed during the current study are available from Prof. M. Nauck upon reasonable request.
