# Supplementary material for: Insulin clearance and incretin hormones following oral and “isoglycemic” intravenous glucose in type 2 diabetes patients under different antidiabetic treatments
Source: Sci Rep. 2022 Feb 15;12:2510. doi: 10.1038/s41598-022-06402-5 (PMC8847358; doi:10.1038/s41598-022-06402-5)
Supplement: Supplementary file 1 — Supplementary Information. [file 41598_2022_6402_MOESM1_ESM.pdf]

Supplementary Figure S1

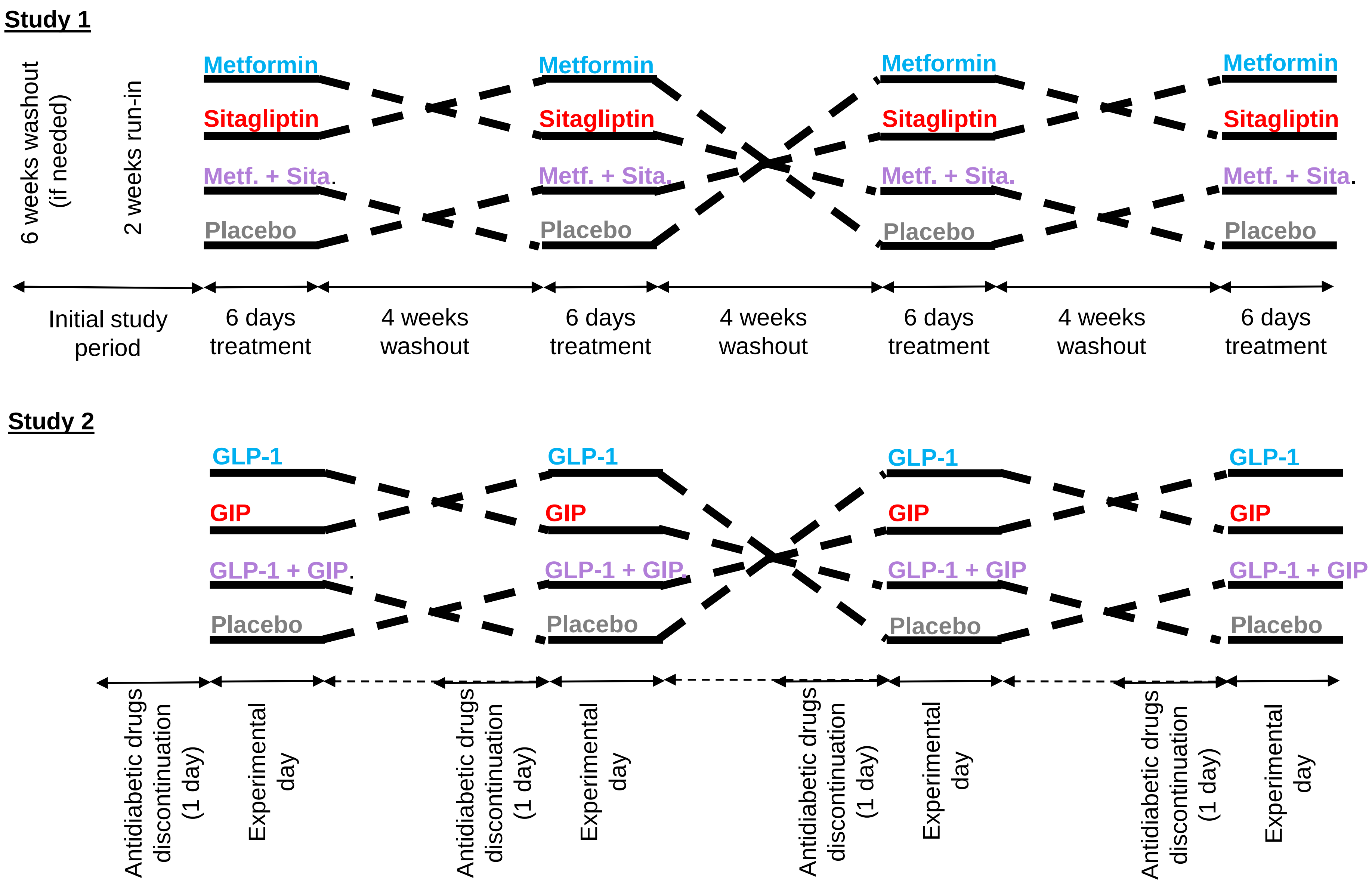

Supplementary Figure S1. Study design for the Study 1 (upper panel) and for the Study 2 (lower panel). The different patients' groups underwent the different treatments or experiments in random order.
